# Characterization of a G-Quadruplex Structure in Pre-miRNA-1229 and in Its Alzheimer’s Disease-Associated Variant rs2291418: Implications for miRNA-1229 Maturation

**DOI:** 10.3390/ijms21030767

**Published:** 2020-01-24

**Authors:** Joshua A. Imperatore, McKenna L. Then, Keefe B. McDougal, Mihaela Rita Mihailescu

**Affiliations:** Department of Chemistry and Biochemistry, Duquesne University, Pittsburgh, PA 15282, USA; imperat1@duq.edu (J.A.I.); thenm@duq.edu (M.L.T.); mcdougal.28@buckeyemail.osu.edu (K.B.M.)

**Keywords:** RNA G-quadruplex, pre-miRNA-1229, Alzheimer’s disease, rs2291418

## Abstract

Alzheimer’s disease (AD), the most common age-related neurodegenerative disease, is associated with various forms of cognitive and functional impairment that worsen with disease progression. AD is typically characterized as a protein misfolding disease, in which abnormal plaques form due to accumulation of tau and β-amyloid (Aβ) proteins. An assortment of proteins is responsible for the processing and trafficking of Aβ, including sortilin-related receptor 1 (SORL1). Recently, a genome-wide association study of microRNA-related variants found that a single nucleotide polymorphism (SNP) rs2291418 within premature microRNA-1229 (pre-miRNA-1229) is significantly associated with AD. Moreover, the levels of the mature miRNA-1229-3p, which has been shown to regulate the SORL1 translation, are increased in the rs2291418 pre-miRNA-1229 variant. In this study we used various biophysical techniques to show that pre-miRNA-1229 forms a G-quadruplex secondary structure that coexists in equilibrium with the canonical hairpin structure, potentially controlling the production of the mature miR-1229-3p, and furthermore, that the AD-associated SNP rs2291418 pre-miR-1229 changes the equilibrium between these structures. Thus, the G-quadruplex structure we identified within pre-miRNA-1229 could potentially act as a novel therapeutic target in AD.

## 1. Introduction

Alzheimer’s disease (AD) is the most common age-related neurodegenerative disease, currently affecting an estimated 4.7 million people in the United States alone, with that number expected to nearly triple to a projected 13.8 million by the year 2050 [1]. The disease is characterized by cognitive declines such as progressive memory loss, confusion, and disorientation, as well as behavioral and mood changes [2]. It is widely accepted that AD is also a leading cause of dementia in older individuals [3,4]. At a molecular level, the development of AD can be associated with the accumulation of filamentous tau tangles inside of neurons and of beta amyloid (Aβ) plaques extracellularly. The accumulation of these proteins interferes with proper neuron-to-neuron communication at synapses in AD, resulting in disrupted communication of information and eventual cell death [5].

Aβ is generated by endoproteolytic cleavage into a 40–42 amino acid fragment from the transmembrane amyloid precursor protein (APP) [6,7]. While the main function of APP has not yet been determined, it is generally agreed upon that dysregulation of its processing is a major factor in the development of the neurotoxic Aβ deposits in AD [8]. Many studies have determined that this may occur due to missense mutations within APP, resulting in an increased production of Aβ_42_, the longer and more amyloidogenic variant that has an increased prevalence in AD patients [9,10]. However, another possibility is that the dysregulation of various other proteins involved in this process may impair proper cleavage and trafficking of APP. Genome-wide association (GWAS) studies have determined a comprehensive list of genes associated with AD, including those that encode for apolipoprotein E (APOE) [11], death-associated protein kinase 1 (DAPK1) [12], interleukin 8 (IL8) [13], transferrin [14], and sortilin-related receptor (SORL1) [15]. The latter of these proteins, SORL1, is a multifunctional membrane-bound receptor protein expressed throughout the brain that plays roles in cell-to-cell signaling and vesicle trafficking [16,17]. Recent studies have reported that SORL1 functions to direct APP into recycling pathways under normal conditions. However, the downregulation of SORL1 expression results in APP being sorted into Aβ-generating pathways, demonstrating a potential mechanistic function of SORL1 in AD pathogenesis [18]. Inherited genetic variants within SORL1 mRNA that are associated with AD have been shown to downregulate SORL1 protein translation [18].

Additionally, other molecular mechanisms, such as microRNA (miRNA) dysregulation, have also been proposed to be involved in AD pathogenesis [19,20]. miRNAs are a class of short, noncoding RNAs approximately 20–24-nucleotides (nt) in length which bind to complementary “seed regions” within the 3′ untranslated region (UTR) of mRNAs and regulate their translation [21]. The mature miRNAs are produced via a distinct biogenesis pathway, which begins with the transcription of miRNA genes to produce the primary miRNA (pri-miRNA), which is further processed by the ribonuclease III enzyme Drosha and the protein DiGeorge syndrome critical region 8 (DGCR8) into precursor miRNAs (pre-miRNAs), 70–90-nt sequences that contain a 2-nt overhang on their 3′ end [22,23,24]. The pre-miRNAs, proposed to fold into extended hairpin structures, are exported to the cytoplasm in a Ran-GTP energy-driven process by the nucleocytoplasmic shuttling protein Exportin-5, which recognizes their 3′ overhang [25]. In the cytoplasm, the endoribonuclease III enzyme Dicer recognizes the pre-miRNA stem-loop structure, cleaving off the terminal loop and yielding an imperfect double-stranded miRNA:miRNA* duplex [26,27]. Dicer further associates with various other proteins to form the RNA-induced silencing complex (RISC), including Argonaute2 (AGO2) and human immunodeficiency virus (HIV) transactivating response RNA (TAR) binding protein (TRBP) [28,29]. While the mature miRNA remains incorporated in the miRNA:RISC complex, the opposite strand, or passenger strand (miRNA*), is discarded [30]. The miRNA-guided RISC complex subsequently recognizes and binds to mRNA targets, leading to mRNA cleavage and/or translational repression.

The importance of regulating miRNA levels has been demonstrated in a multitude of studies, with dysregulated levels being associated with various diseases, such as AD [31], Down syndrome [32], and many cancers [33]. It has been proposed that the formation of alternative secondary structures in pre-miRNAs can affect their processing by Dicer and thus, the mature miRNA production within the cell [34,35]. Bioinformatic studies have concluded that 13–16% of all human pre-miRNA sequences contain guanine-rich (G-rich) regions that have the potential to form G-quadruplex (GQ) structures [34,35]. GQs are non-canonical secondary structures formed by stacking of two or more cyclic, planar G-quartet arrangements of four guanine residues and stabilized by Hoogsteen-type hydrogen bonds, π-π stacking interactions, and intercalating monovalent cations [36,37]. The formation of these GQ structures within G-rich pre-miRNAs has been proposed to inhibit their processing by Dicer due to the lack of a terminal stem loop. Among the pre-miRNAs with G-rich sequences which have the potential to form GQ structures is pre-miRNA-1229, whose mature miRNA product, miRNA-1229-3p, has recently been shown to directly control the expression of SORL1 [38]. Moreover, Ghanbari et al. performed a GWAS which demonstrated that the allelic guanine to adenine rs2291418 variant (cytosine to uracil in the RNA) within pre-miRNA-1229 is associated with AD and that this single nucleotide polymorphism (SNP) enhances the production of miRNA-1229-3p and decreases the levels of SORL1 [38].

In this study, we utilized various biophysical techniques to show that a GQ structure forms in pre-miRNA-1229 which coexists in equilibrium with the canonical extended hairpin structure and that this equilibrium is shifted in the rs2291418 variant, favoring the extended hairpin structure. These results suggest a mechanism leading to the increased production of the mature miRNA-1229-3p in AD and raise the possibility of using the GQ structure within pre-miRNA-1229 as a potential therapeutic target in AD [39].

## 2. Results

### 2.1. The G-Rich Region of Pre-miRNA-1229 Folds into a GQ Structure

**Figure 1:**
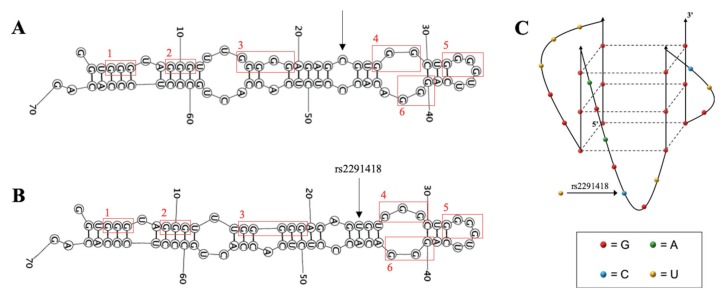
Predicted secondary structures of pre-miRNA-1229 sequences. RNA Structure prediction software (https://rna.urmc.rochester.edu/RNAstructureWeb/) was used to determine the most likely hairpin structures for full-length pre-miRNA-1229_WT (**A**) and pre-miRNA-1229_SNP (**B**). G-tracts are numbered and highlighted by red boxes. The location of the SNP is indicated with an arrow in both structures. The predicted GQ structure of full-length pre-miRNA-1229 was determined using the online QGRS Mapper software (**C**). Nucleotides involved in GQ formation are colored according to the legend below and the location of SNP is indicated with an arrow.

The formation of GQ structures in pre-miRNA sequences has been proposed to alter the efficiency of miRNA production [34,35]. Since pre-miRNA-1229 has multiple G-tracts, numbered 1 to 6 in Figure 1A), we used the online QGRS Mapper (http://bioinformatics.ramapo.edu/ QGRS/analyze.php) software, which predicted that a three-plane GQ structure can form in this pre-miRNA using the G-tracts 2, 3, 4, and 5 (Figure 1A,C). To determine if this G-rich region of pre-miRNA-1229 indeed folds into the predicted GQ structure, we initially investigated a truncated sequence spanning nucleotides 1–42 of pre-miRNA-1229 that contains the six G-tracts that could participate in GQ structure formation (named pre-miRNA-1229_WT GQ, Table 1).

**Figure 2:**
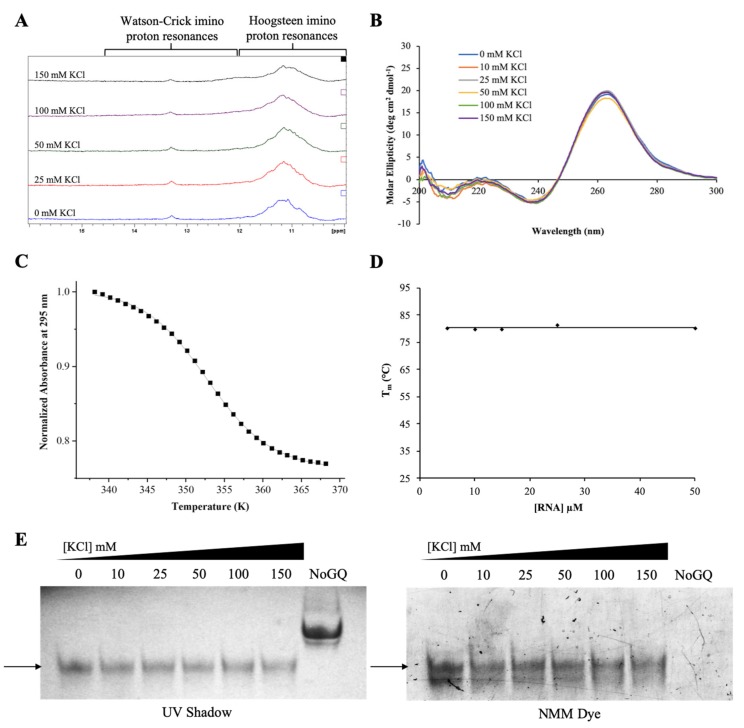
Biophysical characterization of the truncated pre-miRNA-1229_WT GQ sequence. ^1^H NMR spectra (**A**) and CD spectra (**B**) at various KCl concentrations in 10 mM cacodylic acid, pH 6.5, demonstrating the formation of a GQ structure. The UV thermal denaturation hypochromic transition (**C**) at 10 µM RNA in 150 mM KCl was fit using Equation (1) (Materials and Methods), to determine a T_m_ of ~80 °C. The T_m_ values plotted as a function of the RNA concentration (**D**) revealed that an intramolecular GQ structure is formed. Nondenaturing gel electrophoresis (**E**) visualized by UV shadow (left panel) and stained with the GQ-specific *N*-methyl mesoporphyrin IX (NMM) dye (right panel), revealing the formation of a GQ structure at all KCl concentrations.

We first characterized pre-miRNA-1229_WT GQ RNA using 1D ^1^H NMR spectroscopy focusing on the imino proton resonance region between 10 and 15 ppm and titrating increasing KCl concentrations in the range of 0–150 mM, as K^+^ ions stabilize GQ structures (Figure 2A) [36]. Broad resonances are apparent even at 0 mM KCl in the 10–12 ppm region corresponding to guanine imino protons involved in Hoogsteen base pairs in GQ structures [40]. Another resonance is also evident around 13.3 ppm, within the imino proton region 12–14.5 ppm assigned to imino protons involved in Watson–Crick base pairs (Figure 2A, bottom spectrum) [40]. We hypothesize that this resonance arises from Watson–Crick loop–loop interactions within the GQ structure. Increasing KCl concentrations do not seem to have an effect on the intensity of the resonances in the GQ region (Figure 2A), indicating that a GQ structure is stably formed even in the absence of K^+^. RNA sequences have been shown to form stable GQ structures even in the absence of K^+^ ions, while these ions are required for GQ formation in DNA of identical sequence [41].

While both parallel and anti-parallel GQ structures have been observed in DNA, RNA GQs typically only adopt a parallel directionality [42]. To determine the directionality of the GQ structure in pre-miRNA-1229_WT GQ, we performed circular dichroism (CD) spectroscopy experiments. Parallel GQ structures show a positive band at ~265 nm and a negative band at ~240 nm, whereas the signatures of an antiparallel GQ are a positive band at ~295 nm and a negative band at ~260 nm [42]. Our experiments confirm that a parallel GQ forms within pre-miRNA-1229_WT GQ, as we observed a positive band at ~265 nm and a negative one at ~240 nm. Moreover, the intensity of these bands changes minimally as the KCl concentration is increased (Figure 2B), consistent with the ^1^H NMR spectroscopy results (Figure 2A), indicating that the GQ structure formed by pre-miRNA-1229_WT GQ is stable even in the absence of K^+^ ions. Helical structures in A-RNA give rise to a positive band at 260 nm, a negative band at 210 nm, and a small negative CD between 290 and 300 nm [43]. We observed a low intensity negative band at ~210 nm, however, since the intensity of this band is too low with respect to that of the positive band at 260 nm and we did not observe the small negative CD between 290 and 300 nm, we rule out the formation of a hairpin structure in pre-miRNA-1229_WT GQ [43]. Similar to the ^1^H NMR spectroscopy single resonance at 13.3 ppm, we assign the origin of the 210 nm small intensity band to Watson–Crick loop–loop interactions within the GQ structure.

To determine the overall stability of the GQ structure formed in pre-miRNA-1229_WT GQ, we performed UV thermal denaturation experiments at 295 nm, wavelength sensitive to GQ structure dissociation, at a fixed KCl concentration of 150 mM and a range of RNA concentrations from 5 to 50 µM (Supplemental Figure S1A) [44]. At all RNA concentrations investigated, a single hypochromic transition corresponding to the unfolding of a GQ structure was observed (Supplemental Figure S1A). The melting temperature (T_m_) of the GQ structure at each RNA concentration was determined by fitting this hypochromic transition with Equation (1) (Materials and Methods) (Figure 2C) [45] to be ~80 °C at all RNA concentrations investigated (Figure 2D), indicating that pre-miRNA-1229_WT GQ forms an intramolecular GQ structure [46].

Finally, we analyzed pre-miRNA-1229_WT GQ by 20% nondenaturing polyacrylamide gel electrophoresis at various KCl concentrations in the range of 0–150 mM (Figure 2E, full gel shown in Supplemental Figure S1B). A main band was observed at each KCl concentration when the gel was visualized by UV shadowing (Figure 2E, left panel) [47]. This band stained in *N*-methyl mesoporphyrin IX (NMM), a GQ-specific dye (Figure 2E, right panel), revealing that it corresponds to a GQ structure [48]. Additionally, a lower band became clearly visible in the NMM stained gel, indicating the formation of an alternate GQ structure. While Figure 1C depicts the most stable GQ structure predicted by the QGRS Mapper software, multiple GQ structures could be formed in pre-miRNA-1229_WT GQ because it contains six G-tracts and only four of these are required for the formation of a GQ structure [34,35]. A negative control RNA that cannot form a GQ structure (NoGQ) was visible when the gel was visualized by UV shadowing (Figure 2E, left panel), but absent when stained with NMM (Figure 2E, right panel) [47].

Taken together, the results from these biophysical studies confirm the formation of a parallel, intramolecular GQ structure with a T_m_ of ~80 °C in 150 mM KCl in the truncated pre-miRNA-1229_WT GQ sequence.

### 2.2. Full-Length Pre-miRNA-1229 Forms a GQ Structure That Coexists in Equilibrium with an Extended Hairpin Structure

**Figure 3:**
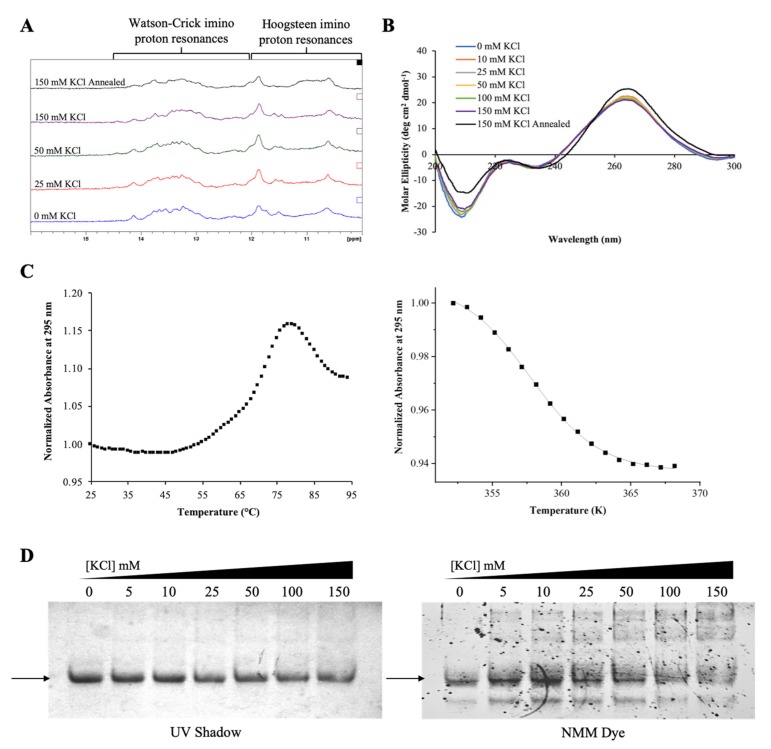
Biophysical characterization of the full-length pre-miRNA-1229_WT FL sequence. ^1^H NMR spectra (**A**) and CD spectra (**B**) at various KCl concentrations in 10 mM cacodylic acid, pH 6.5, reveal an equilibrium between hairpin and GQ structures after annealing the RNA in 150 mM KCl. The UV thermal denaturation hypochromic transition (**C**) at 10 µM RNA in 100 mM KCl was fit using Equation (1) (Materials and Methods), to determine a T_m_ of ~85 °C. Nondenaturing gel electrophoresis (**D**) visualized by UV shadow (left panel) and stained with the GQ-specific NMM dye, revealing the formation of multiple GQ structures.

Next we investigated if the GQ structure formed by the G-rich region of pre-miR-1229 is retained within the full-length pre-miRNA-1229_WT sequence (pre-miRNA-1229_WT FL, Table 1), where additional nucleotides past position 42 compete for the formation of the canonical extended hairpin structure. 1D ^1^H NMR spectroscopy experiments were performed to monitor secondary structure formation by observing the imino proton resonance region while titrating increasing KCl concentrations (Figure 3A). Multiple resonances between 12 and 14.5 ppm, which indicate the formation of a hairpin structure, were observed for pre-miRNA-1229_WT FL, these resonances being absent in pre-miRNA-1229_WT GQ (compare Figure 2A and Figure 3A) [40]. The broadness of these resonances could be due to the size of the RNA investigated, but it could also indicate possible exchange between different conformations. Sharper resonances on a broad envelope background were also observed in the range of 10–12 ppm. As discussed above, resonances in the range of 10–12 ppm are assigned to guanine imino protons involved in Hoogsteen base pairs in GQ structures [40]. However, in sequences that cannot form GQ structures, sharper resonances in these regions were assigned to GU wobble base pairs or side-by-side GG/GA base pairs, while broader resonances were assigned to unpaired G or U imino protons located in hairpin loops [49,50]. Moreover, although Watson–Crick imino protons are observed typically in the range of 12–14.5 ppm, there are instances where the imino proton of a guanine involved in a GC base pair immediate to an internal loop could give rise to a resonance around 11.9 ppm [49]. The predicted extended hairpin structure of pre-miRNA-1229_WT FL (Figure 1A) contains internal loops where GG or GA base pairs could form, unpaired G and U residues in the terminal loop, as well as GC Watson–Crick base pairs immediately adjacent to internal loops, all of which could give rise to the sharper resonances observed in the 10–12 ppm region. The presence of a broader envelope resonance centered around 11 ppm, visible especially upon addition of higher KCl concentrations, suggests also the presence of a GQ structure (Figure 3A). However, the GQ structure signatures become clearly visible only once the sample is annealed in the presence of 150 mM KCl, as the intensity of the broad resonance around 11 ppm increases with the concomitant decrease of the resonances in the Watson–Crick imino proton region (Figure 3A, top spectrum). These results indicate that pre-miRNA-1229_WT FL forms a GQ structure coexisting in equilibrium with a hairpin structure, with the GQ structure being stabilized by annealing the RNA in the presence of KCl.

CD spectroscopy experiments were performed next for pre-miRNA-1229_WT FL at varying KCl concentrations in the range of 0–150 mM (Figure 3B), revealing a positive band at ~265 nm, a negative band at ~235 nm, an intense negative band at 210 nm, and a small negative CD between 290 and 300 nm. These bands, which did not change significantly with the KCl titrations, are consistent with the presence of both a hairpin and a GQ structure coexisting in equilibrium [42,43]. Additionally, consistent with ^1^H NMR spectroscopy results (Figure 3A, top spectrum), only when the sample was annealed at 95 °C in 150 mM KCl was a noticeable difference observed in the CD spectrum, with an increase in the intensity of the positive band at ~265 nm and a decrease in the intensity of the negative band at ~210 nm, indicating a shift of the equilibrium towards the GQ structure.

UV thermal denaturation experiments were performed at 295 nm to determine the stability of the GQ structure formed within the pre-miRNA-1229_WT FL sequence [44]. A hypochromic transition corresponding to GQ structure dissociation was observed between 79 and 95 °C, and a hyperchromic transition was observed between 46 and 79 °C, corresponding to the hairpin structure dissociation (Figure 3C). These experiments were performed in the presence of 100 mM KCl, as at 150 mM KCl, the GQ structure was too stable, resulting in an incomplete hypochromic transition (data not shown). The GQ dissociation transition was fit using Equation (1) (Materials and Methods) (Figure 3C) to determine a T_m_ of ~85 °C [45].

To further characterize the equilibrium between the hairpin and GQ structures in pre-miRNA-1229_WT FL, we performed 15% nondenaturing gel electrophoresis at various concentrations of KCl (Figure 3D). When the gel was visualized by UV shadowing, a single main band was evident at all concentrations of KCl, with very faint upper bands also apparent (Figure 3D, left panel) [47]. When the gel was stained in the GQ-specific NMM dye, the main band stained, but multiple upper bands and a lower band also became clearly visible (Figure 3D, right panel), indicating the presence of multiple GQ structure conformations due to the presence of six G-tracts in the sequence (Figure 1A), as well as possible stacking interactions between GQ structures [48].

The results from these biophysical characterization studies show that GQ structures are retained in the context of the full-length pre-miRNA-1229_WT FL sequence, which coexist in equilibrium with the canonical extended hairpin structure. Additionally, while the GQ structure can form in the absence of K^+^ ions, it is stabilized by annealing the RNA in the presence of 150 mM KCl (Figure 3A).

**Figure 4:**
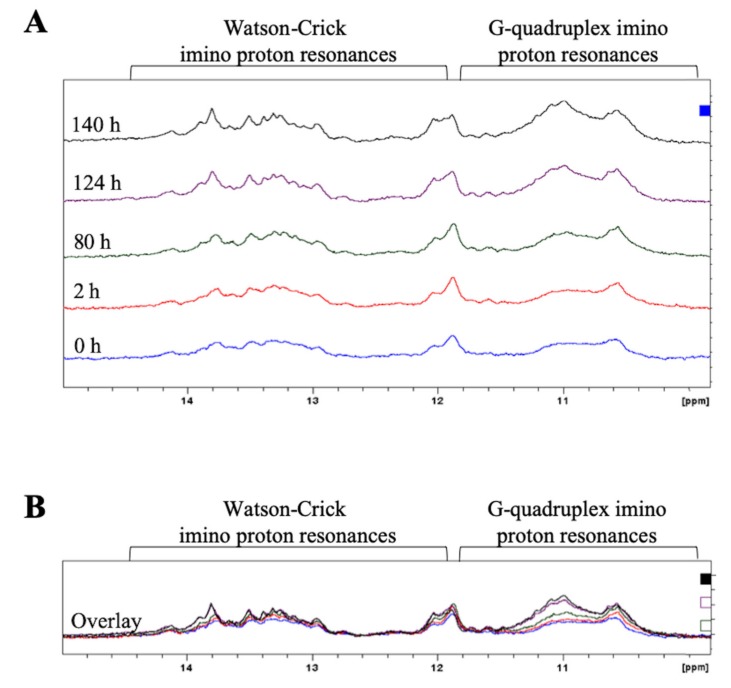
Time-dependent ^1^H NMR spectra of full-length pre-miR-1229_WT FL in the presence of 150 mM KCl and 1 mM MgCl_2_ (**A**). After approximately 140 h, the GQ structure is the preferred structure, as indicated by the increased intensity of the imino proton resonances corresponding to Hoogsteen base pairs in the GQ structure compared to those assigned to Watson–Crick imino proton resonances. An overlay of the spectra is shown in (**B**).

To monitor the equilibrium between the GQ and hairpin structures in ionic conditions closer to physiological conditions, 1 mM MgCl_2_ was added to the NMR sample previously annealed in the presence of 150 mM KCl (Figure 4A, bottom spectrum), acquiring time-dependent spectra over the course of 6 days, during which time the sample was incubated at 37 °C (Figure 4). Mg^2+^ ions stabilize various RNA structures, including hairpins, and this was observed for pre-miRNA-1229_WT FL since the imino proton resonances corresponding to Watson–Crick base pairs become sharper over time (Figure 4A) [40,51]. However, it is interesting to note that the broad resonances centered around 11 ppm, corresponding to guanine imino protons engaged in Hoogsteen base pairs in the GQ structure, increase in intensity, suggesting that the equilibrium is shifted towards the GQ structure over time (Figure 4A and overlay of spectra in Figure 4B).

Taken together, these studies show for the first time that a GQ structure coexists in equilibrium with a hairpin structure within the G-rich pre-miRNA-1229 sequence. GQ structure formation within pre-miRNAs has been previously shown to reduce the levels of mature miRNA production [34,35], thus, our results suggest that the GQ structure within pre-miRNA-1229 could provide a fine-tuning control mechanism for the production of mature miRNA-1229. The results of this study have implications for AD, as the mature miRNA-1229 has been shown to regulate the translation of SORL1, a protein whose downregulated expression has been shown to result in APP being sorted into Aβ-generating pathways [18,38]. Thus, the GQ structure we characterized within pre-miRNA-1229 could potentially become a therapeutic target in AD, as molecules that stabilize it could ultimately reduce the levels of mature miRNA-1229 [39].

### 2.3. The GQ Structure Formed by the G-Rich Region of Pre-miRNA-1229 Is Retained in the AD-Associated rs2291418 Variant

**Figure 5:**
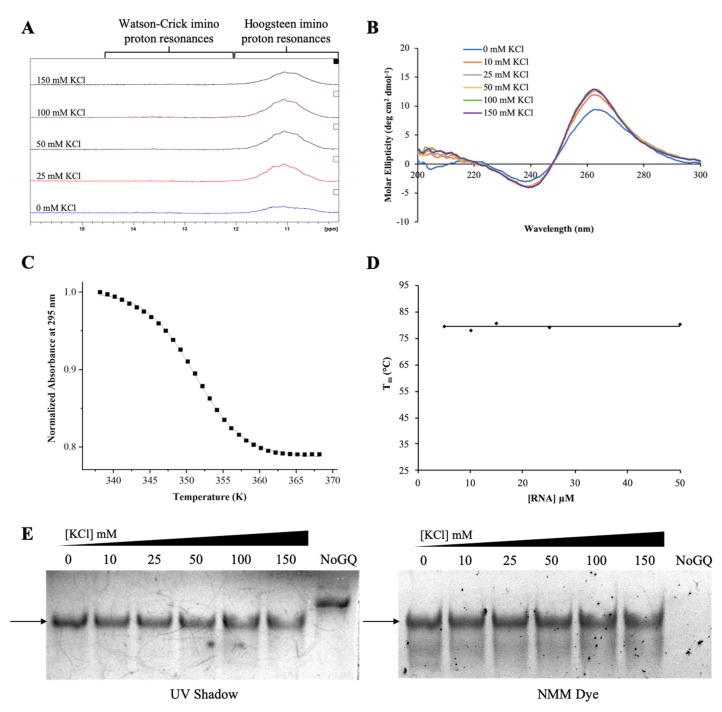
Biophysical characterization of the truncated pre-miR-1229_SNP GQ sequence. ^1^H NMR spectra (**A**) and CD spectra (**B**) at various KCl concentrations in 10 mM cacodylic acid, pH 6.5, demonstrating the formation of a GQ structure. The UV thermal denaturation hypochromic transition (**C**) at 10 µM RNA in 150 mM KCl was fit using Equation (1) (Materials and Methods), to determine a T_m_ of ~78 °C. The T_m_ values plotted as a function of the RNA concentration (**D**) revealed that an intramolecular GQ structure is formed. Nondenaturing gel electrophoresis (**E**) visualized by UV shadow (left panel) and stained with the GQ-specific NMM dye (right panel), revealing the formation of a GQ structure at all KCl concentrations.

Ghanbari et al. demonstrated not only that miRNA-1229 is implicated in AD by regulating the translation of SORL1, but also that the pre-miRNA-1229 variant rs2291418 (cytosine to uracil in the RNA, indicated by arrows in Figure 1), which results in increased levels of mature miRNA-1229-3p, is associated with AD [38]. Given that the rs2291418 SNP is located within the G-rich region of pre-miRNA-1229 (arrows in Figure 1A–C), we hypothesized that this variant could potentially change the equilibrium between the GQ and hairpin structures within pre-miRNA-1229 and consequently dysregulate the control of the mature miRNA-1229 production. Similar to our initial investigation of the truncated 42-nt pre-miRNA-1229_WT GQ, we generated a truncated pre-miRNA-1229_SNP sequence (named pre-miRNA-1229_SNP GQ, Table 1) and characterized it by using similar biophysical techniques. ^1^H NMR spectroscopy experiments of pre-miRNA-1229_SNP GQ revealed broad imino proton resonances centered around 11 ppm which increased in intensity as KCl was titrated in the sample (Figure 5A), indicative of the formation of a GQ structure that is stabilized by K^+^ ions [40]. Interestingly, no resonances were observed in the region corresponding to Watson–Crick imino protons as was observed for the truncated WT sequence (compare Figure 2A and Figure 5A), suggesting that the rs2291418 SNP disrupts the loop–loop interactions that were occurring within the wild-type sequence.

The directionality of the GQ structure formed by pre-miRNA-1229_SNP GQ was analyzed using CD spectroscopy (Figure 5B) while titrating KCl in the range of 0–150 mM. While the signature positive band at ~265 nm and negative band at ~240 nm corresponding to a parallel GQ are evident at all concentrations of KCl, a noticeable increase in band intensity is observed upon the addition of 10 mM KCl, indicating the further stabilization of this GQ structure by K^+^ ions and consistent with the ^1^H NMR spectroscopy results (Figure 5A) [42]. These results differ from the pre-miRNA-1229_WT GQ results (compare Figure 2B and Figure 5B), as the GQ formed within that sequence was stable even in the absence of K^+^ ions. Moreover, the small intensity negative band at 210 nm observed for pre-miRNA-1229_WT GQ (Figure 2B) is absent from the spectra of pre-miRNA-1229_SNP GQ (Figure 5B), once again suggesting the absence of loop–loop interactions within the GQ structure formed within this sequence and consistent with the ^1^H NMR spectroscopy results.

To determine the stability of the GQ structure formed by pre-miRNA-1229_SNP GQ, UV thermal denaturation experiments were performed at a fixed KCl concentration of 150 mM and RNA concentrations in the range of 5–50 µM (Supplemental Figure S3A). At all RNA concentrations, a single hypochromic transition corresponding to GQ structure dissociation was observed which was fit (Figure 5C) using Equation (1) (Materials and Methods) to determine an average T_m_ of ~78 °C [45]. As with the wild-type pre-miRNA-1229_WT GQ sequence, the GQ melting temperatures do not depend on the RNA concentration, indicating that pre-miRNA-1229_SNP GQ forms an intramolecular GQ structure (Figure 5D) [46]. The T_m_ values of the GQ structures formed by the wild-type and SNP truncated sequences of pre-miRNA-1229 are almost identical at 150 mM KCl, suggesting that the additional stabilization observed for the GQ structure formed by pre-miRNA-1229_WT GQ originates from loop–loop interactions, potentially involving the mutated cytosine to uracil residue, interactions that are absent in pre-miRNA-1229_SNP GQ.

Finally, 20% nondenaturing gel electrophoresis (Figure 5E) was performed at various KCl concentrations in the range of 0–150 mM. At each KCl concentration, a single main band was evident when the gel was visualized by UV shadowing (Figure 5E, left panel) [47]. This band was also present when the gel was stained by NMM (Figure 5E, right panel), indicating that it corresponds to a GQ structure [48]. An additional lower band was also present in the NMM stained gel, indicating the formation of alternative GQ structures, as was seen with pre-miRNA-1229_WT GQ (Figure 2E).

Taken together, these results show that a parallel, intramolecular GQ structure forms within pre-miRNA-1229_SNP GQ, but this structure lacks the loop–loop interactions that stabilize the GQ structure formed within the wild-type pre-miRNA-1229_WT GQ sequence.

### 2.4. The AD-Associated rs2291418 Mutation within the Full-Length Pre-miRNA-1229 Shifts the Equilibrium from the GQ Structure to the Extended Hairpin Structure

**Figure 6:**
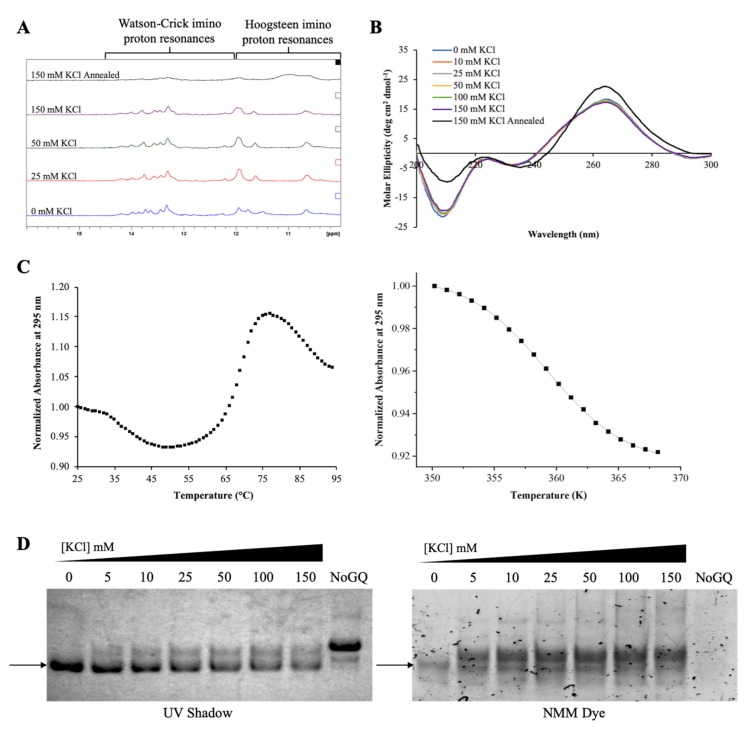
Biophysical characterization of the full-length pre-miR-1229_SNP FL sequence. ^1^H NMR spectra (**A**) and CD spectra (**B**) at various KCl concentrations in 10 mM cacodylic acid, pH 6.5 reveal an equilibrium between hairpin and GQ structures after annealing the RNA in 150 mM KCl. UV thermal denaturation hypochromic transition (**C**) at 10 µM RNA in 100 mM KCl was fit using Equation (1) (Materials and Methods), to determine a T_m_ of ~86 °C. Nondenaturing gel electrophoresis (**D**) visualized by UV shadow (left panel) and stained with the GQ-specific NMM dye (right panel), revealing the formation of multiple GQ structures as well as a hairpin structure.

To determine if the GQ structure is retained in the full-length rs2291418 pre-miRNA-1229 sequence, we characterized the full 70-nucleotide sequence (named pre-miRNA-1229_SNP FL, Table 1). Similar to the full-length WT sequence, ^1^H NMR spectroscopy experiments (Figure 6A, bottom spectrum) revealed imino proton resonances in both the GQ and Watson–Crick imino proton regions [40]. The resonances in the Watson–Crick region are well defined and sharper than those observed for pre-miRNA-1229_WT FL at all concentrations (compare Figure 3A and Figure 6A), indicating a stable hairpin structure formation. We also observed sharper resonances in the 10–12 ppm region (Figure 6A) that could originate from GG base pairs within internal loops, unpaired G and U residues present in the terminal loop, as well as GC Watson–Crick base pairs adjacent to internal loops of pre-miRNA-1229_SNP FL (Figure 1B) [49,50]. However, in contrast to the spectra of pre-miRNA-1229_WT FL, these resonances have a completely flat baseline even at 150 mM KCl concentrations. Only when the sample was annealed at 150 mM KCl do broad envelope resonances appear clearly in the 10–12 pm region, with the concomitant decrease in intensity of the Watson–Crick resonances, indicating that a GQ structure does not stably form in pre-miRNA-1229_SNP FL until the sample is annealed in the presence of K^+^ ions.

To determine the directionality of the GQ structure formed in pre-miRNA-1229_SNP FL, CD spectroscopy experiments were performed at KCl concentrations in the range of 0–150 mM (Figure 6B). We observed a positive band at ~265 nm, a negative band at ~235 nm, a negative band at ~210 nm, and a small negative CD between 290 and 300 nm, consistent with the presence of a hairpin structure [42,43]. We cannot rule out the presence of a GQ structure based solely on the CD data since the signature bands of a parallel GQ structure overlap to some extent with those of an A-type hairpin structure [43]. However, we noted that the annealing of the sample in the presence of 150 mM KCl caused an increase of the intensity of the positive band at ~265 nm with a concomitant decrease of the intensity of the negative band at ~210 nm and a shift of the negative band from 235 to 240 nm, indicating the formation of a stable GQ structure which coexists with the hairpin structure. These results are similar to those from the ^1^H NMR spectroscopy experiments which show that a stable GQ structure does not form in pre-miRNA-1229_SNP FL until the sample is annealed in the presence of K^+^ ions.

UV thermal denaturation experiments were performed (Figure 6C) at 100 mM KCl to determine the stability of the GQ structure formed within pre-miRNA-1229_SNP FL. Similar to pre-miRNA-1229_WT FL, a hyperchromic transition corresponding to hairpin dissociation and a hypochromic transition corresponding to the GQ structure unfolding were observed (Figure 6C, left). The hypochromic transition was fit with Equation (1) (Materials and Methods) to determine a T_m_ of ~86 °C (Figure 6C, right), which is comparable with the T_m_ of the GQ structure formed by pre-miRNA-1229_WT FL [45].

Finally, 15% nondenaturing gel electrophoresis was performed at KCl concentrations in the range of 0–150 mM (Figure 6D). When the gel was visualized by UV shadowing (Figure 6D, left panel), a single main band was observed at 0 mM KCl, whereas a second, upper band is clearly visible with the addition of just 5 mM KCl (compare Figure 6D lanes 1 and 2) [47]. As the concentration of KCl in the sample increased, the primary band seen at 0 mM KCl decreased in intensity, indicating an equilibrium between competing structures. When the gel was stained in NMM (Figure 6D, right panel), the main band clearly observed at 0 mM KCl by UV shadowing stains faintly, indicating that a GQ structure forms [48]. Since all the RNA samples were annealed prior to their loading on the gel, it is possible that the annealing could have promoted formation of the GQ structures even at 0 mM KCl. Upon the addition of increasing KCl concentrations, the additional upper band observed when the gel was visualized by UV shadowing also stains in NMM, indicating that it originates from a GQ structure (Figure 6E, right panel). Additional fainter upper bands and a lower band are also apparent in the NMM stained gel, indicating alternative GQ structures and/or GQ stacking interactions, as was observed with pre-miRNA-1229_WT FL (Figure 3E, right panel). These results confirm that pre-miRNA-1229_SNP FL exists in equilibrium between a hairpin and GQ structures with the GQ conformations being stabilized when the RNA was annealed at 150 mM KCl.

**Figure 7:**
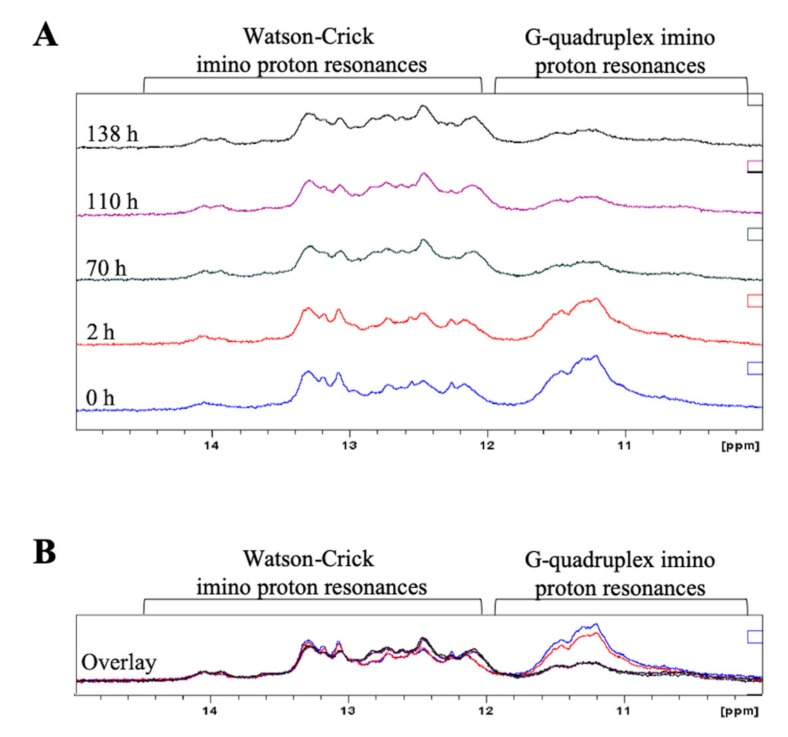
Time-dependent NMR spectra of full-length pre-miR-1229_SNP FL in the presence of 150 mM KCl and 1 mM MgCl_2_ (**A**). After 138 h, the hairpin structure is the preferred structure, as noted by the increased intensity of the Watson–Crick imino proton resonances compared to the decreased intensity of the imino proton resonances assigned to Hoogsteen base pairs in the GQ structure. An overlay of the spectra is shown in (**B**).

To monitor the equilibrium between the GQ and hairpin structure in pre-miRNA-1229_SNP FL in conditions that stabilize the hairpin conformation, time-dependent ^1^H NMR spectroscopy experiments were performed in the presence of 150 mM KCl and 1 mM MgCl_2_ (Figure 7A), while the sample was incubated at 37 °C. In the presence of Mg^2+^ ions, there is a stabilization of the hairpin structure reflected by sharper Watson–Crick imino proton resonances between 12 and 14.5 pm [40], but strikingly, the GQ broad imino proton resonances centered around 11 ppm decreased drastically over time (Figure 7A and overlay of spectra in Figure 7B), indicating that the equilibrium shifted towards the hairpin structure. These results are in stark contrast to the time-dependent ^1^H NMR spectroscopy experiments for pre-miRNA-1229_WT FL (compare to Figure 4) in which the equilibrium shifted over time towards the GQ structure.

Our results indicate that while a hairpin and GQ structure coexist within the pre-miRNA-1229_SNP FL sequence, the hairpin structure is ultimately favored in the rs2291418 AD-associated variant.

## 3. Discussion

Ghanbari et al. proposed that the observed increased levels of miRNA-1229-3p in the AD-associated rs2291418 SNP within pre-miRNA-1229 are due to the stabilization of the hairpin structure by the SNP. They predicted that the minimum free energy of the SNP hairpin was ΔG° = −36.10 kcal/mol, whereas that of the WT was ΔG° = −31.90 kcal/mol [38]. However, when we folded the pre-miRNA-1229_WT FL and pre-miRNA-1229_SNP FL with the RNA Structure software (Figure 1A,C), the two hairpin structures were predicted to have free energies of ΔG° = −36.0 kcal/mol and ΔG° = −36.7 kcal/mol, respectively, which differ only by 0.7 kcal/mol. In this study, we showed that both the wild-type and SNP pre-miRNA-1229 sequences form GQ structures that coexist in equilibrium with the canonical extended hairpin structure and that the GQ structure is destabilized in the rs2291418 variant, possibly due to the lack of loop–loop interactions, resulting in a shift in equilibrium towards the hairpin structure.

Investigating the molecular pathways and key mechanistic steps involved in AD pathogenesis is vital for the development of therapeutic interventions for the disease. Research in the field has shifted focus to the involvement of noncoding miRNAs in AD due to their contributions in regulating gene expression, Aβ and tau protein maintenance, inflammation, and cell death [19,20]. A recent study by Ke et al. suggested that miRNA-107 plays a neuroprotective role in AD progression and that its expression levels are reduced in an Aβ-induced AD model [52]. Furthermore, the authors of this study propose a mechanism in which the long noncoding RNA (lncRNA) NEAT1 binds to miRNA-107, leading to this downregulation. In this study, we investigated pre-miRNA-1229 due to the association of the rs2291418 variant with AD and subsequent upregulation of miRNA-1229-3p production, and we identified a GQ structure present in both the wild-type and rs2291418 variant which has the potential to be a new therapeutic target in AD [38,39].

However, the results of this study have implications beyond the AD context, as multiple other studies have determined that the upregulation of miRNA-1229 can be associated with various other diseases. Expression levels of miRNA-1229 were found to be correlated with increased severity of coronary artery calcification and increased tumor size in colorectal cancer [53,54]. Furthermore, miRNA-1229 overexpression has been proposed to promote cell proliferation and tumorigenicity in breast cancer [55].

This study proposes a mechanism in which the pre-miRNA-1229 equilibrium between GQ and extended hairpin structures is altered in the rs2291418 variant, potentially explaining the increased mature miRNA-1229-3p production observed in the AD-linked variant. These results warrant further investigation into how RNA structure can have downstream effects in disease pathogenesis, and ultimately how GQ structures, such as those found in pre-miRNA-1229, could be therapeutically targeted for intervention purposes in the future.

## 4. Materials and Methods

### 4.1. In Vitro RNA Synthesis

Pre-miRNA sequences (Table 1) were produced by in vitro transcription reactions using T7 RNA polymerase (synthesized in-house), following the procedure by Milligan and Uhlenbeck [56]. The DNA templates for each sequence were chemically synthesized by TriLink Biotechnologies, Inc (San Diego, CA, USA). The RNA oligonucleotides were purified by 20% 8 M urea denaturing polyacrylamide gel electrophoresis (PAGE) and electrophoretic elution, followed by extensive dialysis against 10 mM cacodylic acid, pH 6.5. The concentration of each oligonucleotide was measured using a NanoDrop 2000 UV-Vis spectrophotometer (ThermoFisher Scientific, Waltham, MA, USA) and their purity was checked by denaturing gel electrophoresis (data not shown).

### 4.2. ^1^H NMR Spectroscopy

One-dimensional (1D) proton (^1^H) NMR spectroscopy experiments were performed on a 500 MHz Bruker AVANCE spectrometer (Bruker Corporation, Billerica, MA, USA) at 25 °C using Topspin 3.2 software. Water suppression was carried out by using the Watergate pulse sequence [57]. Pre-miRNA samples with concentrations ranging from 100 to 300 µM were prepared in a volume of 250 μL 10 mM cacodylic acid, pH 6.5, in a 90:10 ratio of H_2_O:D_2_O. GQ formation was observed by titrating increasing concentrations of KCl from a 2 M stock solution to each sample. The samples containing 150 mM KCl were also annealed by boiling at 95 °C for 5 min and cooling on the bench for 15 min. Time-dependent NMR spectroscopy studies were conducted following the addition of 1 mM MgCl_2_ to each sample and incubating at 37 °C, while acquiring spectra over time.

### 4.3. Circular Dichroism Spectroscopy

Circular dichroism (CD) spectroscopy experiments were recorded on a Jasco J-810 spectropolarimeter (JASCO, Easton, MD, USA) at 25 °C. Pre-miRNA samples were prepared in 10 mM cacodylic acid, pH 6.5 to a final RNA concentration of 10 μM in a volume of 200 µL. GQ formation was monitored by titrating increasing amounts of KCl from a 2 M stock solution to each sample and subsequently annealing the sample at 95 °C for 5 min once a final KCl concentration of 150 mM was reached. Spectra were measured between 200 and 300 nm and corrected for solvent contributions. Each spectrum was scanned seven times with a 1 s response time and a 2 nm bandwidth. CD spectroscopy experiments were performed in duplicate (*n* = 2) for each RNA.

### 4.4. UV Spectroscopy Thermal Denaturation

Thermal denaturation experiments were conducted using a Varian Cary 3E UV/Vis spectrophotometer (Agilent, Santa Clara, CA, USA) equipped with a Peltier cell. RNA samples were annealed in 10 mM cacodylic acid, pH 6.5, containing 100–150 mM KCl. Samples were heated from 25 to 95 °C at a rate of 0.2 °C min^−1^, recording points every 1 °C at 295 nm, a wavelength previously identified to be sensitive to G-quadruplex dissociation [44]. To determine if the structures fold into intermolecular or intramolecular conformations, the melting temperatures of the G-quadruplex structures were determined at various RNA concentrations. The transitions of each dissociation were identified and fit with Equation (1) [45]:

(1) AT= AU+AFe−ΔH°RTeΔS°Re−ΔH°RTeΔS°R+1,

where *A_U_* and *A_F_* represent the absorbance of the unfolded and native GQ RNA, respectively, and *R* is the universal gas constant.

### 4.5. Nondenaturing Polyacrylamide Gel Electrophoresis

RNA samples were prepared with varying KCl concentrations and a constant RNA concentration of 10 μM, followed by heating for 5 min at 95 °C and slow cooling to room temperature. Samples were run on 15% or 20% acrylamide gels and visualized using an AlphaImager (ProteinSimple, San Jose, CA, USA) by UV shadowing at 254 nm [47]. Gels were subsequently stained in *N*-methyl mesoporphyrin IX (NMM), a GQ-specific fluorescent probe, and visualized to determine if the GQ structure forms at various concentrations of KCl [48]. Gel electrophoresis experiments were performed in triplicate (*n* = 3) for each RNA.

## 5. Conclusions

Collectively, the biophysical characterization experiments performed in this study determined that pre-miRNA-1229 forms a GQ structure which coexists in equilibrium with an extended hairpin structure. This study adds to the growing number of G-rich pre-miRNA sequences shown to form GQ structures that could interfere with their processing by Dicer and the production of mature miRNA, ultimately providing a fine-tuning mechanism for regulating translation. Furthermore, we show that while in the WT pre-miRNA-1229 the GQ structure is preferred, in the AD-associated rs2291418 pre-miRNA-1229 variant the equilibrium is shifted towards the extended hairpin structure which can be processed by Dicer, possibly explaining the observed increased production of the mature miRNA-1229-3p in this variant. The results from this study elucidate a potential role of RNA secondary structure equilibrium in the AD pathogenesis and warrant further investigation into how GQ structures could be therapeutically targeted in the future.

## Figures and Tables

**Table 1:** RNA sequences used in this study. G-rich areas within pre-miRNA sequences with potential to form G-quadruplex structures are underlined. The SNP mutation locations are highlighted in bold and italic.

pre-miRNA-1229_WT GQ	5′ GGGUAGGGUUUGGGGGAGAG***C***GUGGGCUGGGGUUCAGGG ACA 3′
pre-miRNA-1229_WT FL	5′ GGUGGGUAGGGUUUGGGGGAGAG***C***GUGGGCUGGGG UUCAGGGACACCCUCUCACCACUGCCCUCCCACAG 3′
pre-miRNA-1229_SNP GQ	5′ GGGUAGGGUUUGGGGGAGAG***U***GUGGGCUGGGGUUCAGGG ACA 3′
pre-miRNA-1229_SNP FL	5′ GGUGGGUAGGGUUUGGGGGAGAG***U***GUGGGCUGGGG UUCAGGGACACCCUCUCACCACUGCCCUCCCACAG 3′

## Supplementary Materials

Supplementary materials can be found at https://www.mdpi.com/1422-0067/21/3/767/s1.

## Abbreviations

AD	Alzheimer’s disease
Aβ	β-amyloid
SORL1	Sortilin-related receptor
miRNA	microRNA
nt	nucleotide
RISC	RNA-induced silencing complex
GQ	G-quadruplex
SNP	Single nucleotide polymorphism
NMR	Nuclear magnetic resonance
CD	Circular dichroism
UV	Ultraviolet
T_m_	Melting temperature
NMM	*N*-methyl mesoporphyrin IX
