# Supplementary material for: Characterization of a G-Quadruplex Structure in Pre-miRNA-1229 and in Its Alzheimer’s Disease-Associated Variant rs2291418: Implications for miRNA-1229 Maturation
Source: Int J Mol Sci. 2020 Jan 24;21(3):767. doi: 10.3390/ijms21030767 (PMC7037302; doi:10.3390/ijms21030767)

Supplemental Figure S1. Biophysical characterization of pre-miRNA-1229_WT GQ. UV thermal denaturation profiles **(A)** of pre-miRNA-1229_GQ WT reveal a hypochromic transition, corresponding to GQ dissociation, between 65°C and 95°C. The average T_m was determined to be ~80°C. Full 20% non-denaturing gels **(B)** are shown, visualized with UV shadow (left panel) and stained in NMM (right panel).

Supplemental Figure S2. Biophysical characterization of pre-miRNA-1229_WT FL. Full 15% nondenaturing gels of pre-miRNA-1229_WT FL are shown, visualized with UV shadow (left panel) and stained in NMM (right panel).

Supplemental Figure S3. Biophysical characterization of pre-miRNA-1229_SNP GQ. UV thermal denaturation profiles **(A)** of pre-miRNA-1229_GQ SNP reveal a hypochromic transition, corresponding to GQ dissociation, between 65°C and 95°C. The average T_m was determined to be ~79°C. Full 20% non-denaturing gels **(B)** are shown, visualized with UV shadow (left panel) and stained in NMM (right panel).

Supplemental Figure S4. Biophysical characterization of pre-miRNA-1229_SNP FL. Full 15% non-denaturing gels of pre-miRNA-1229_SNP FL are shown, visualized with UV shadow (left panel) and stained in NMM (right panel).

Supplemental Figure S1

A

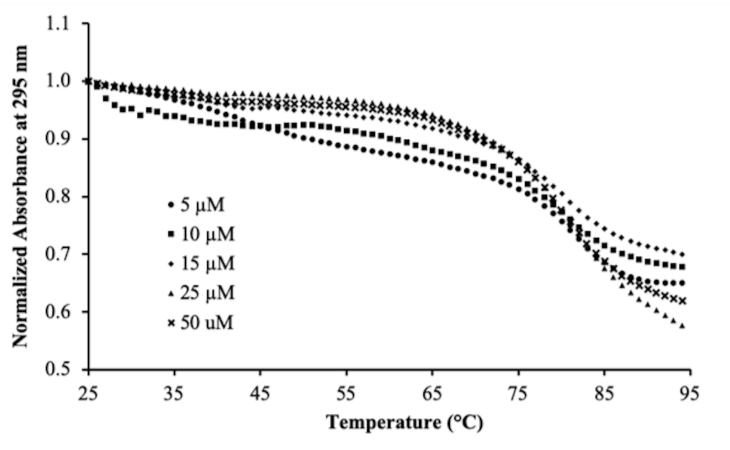

B

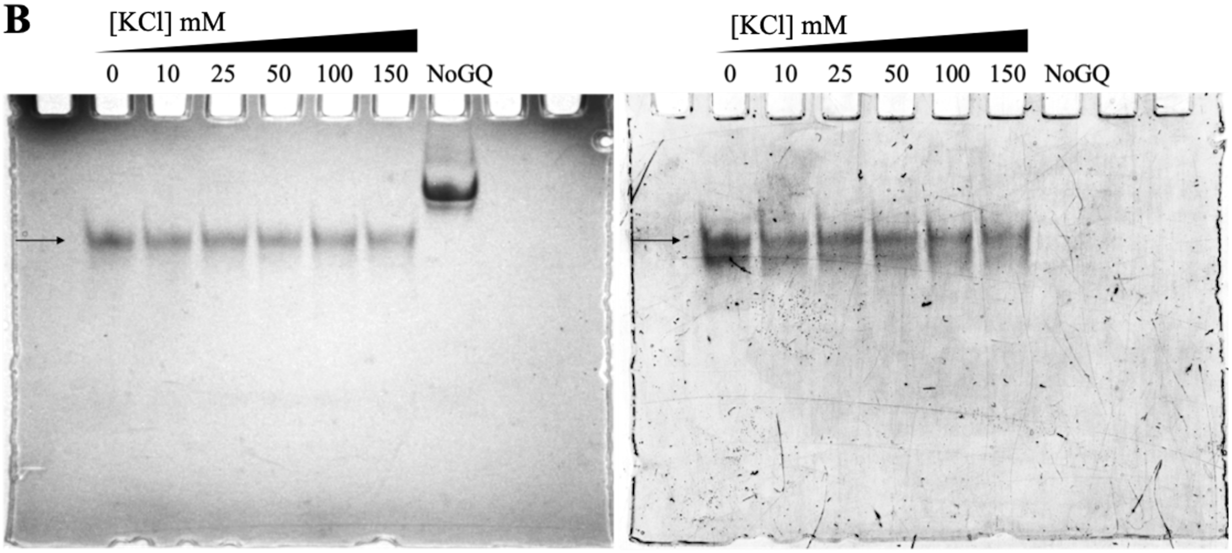

UV Shadow

NMM Dye

Supplemental Figure S2

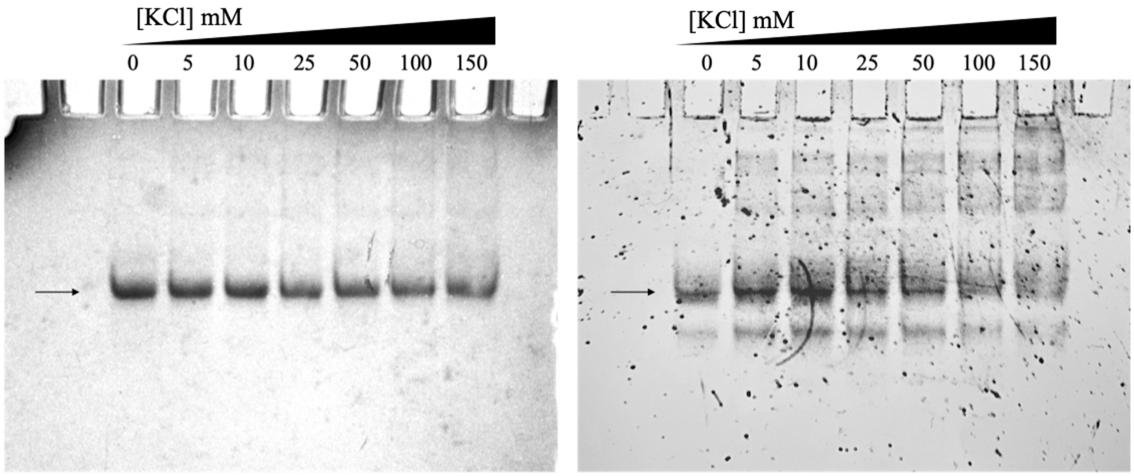

UV Shadow

NMM Dye

Supplemental Figure S3

A

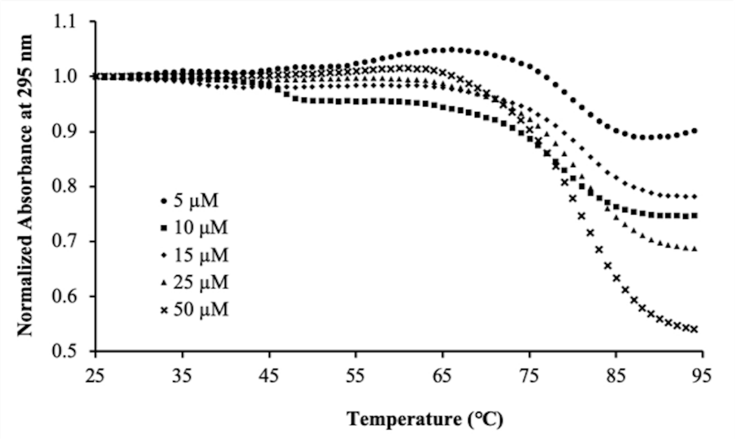

B

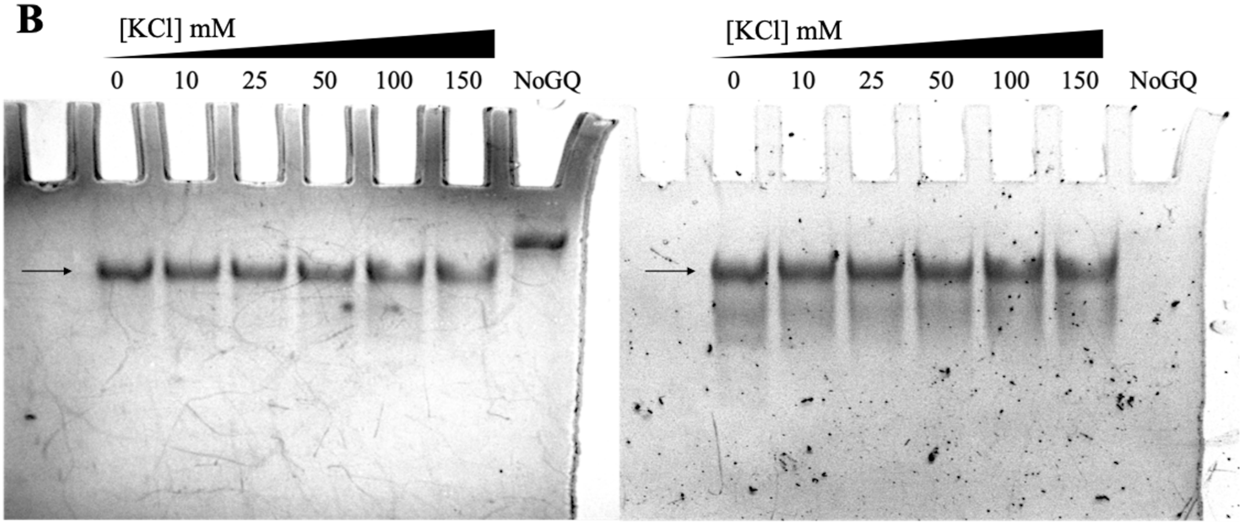

Supplemental Figure S4

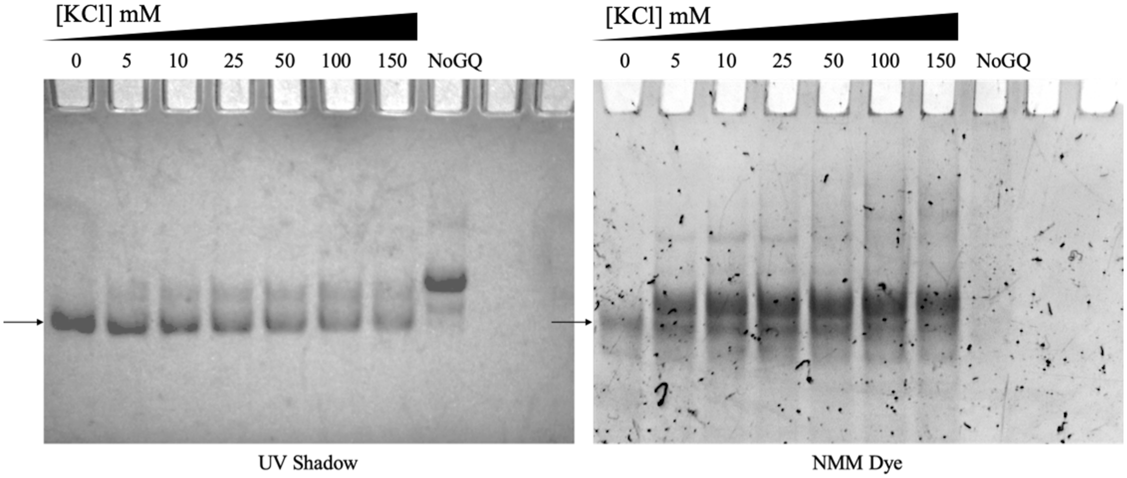

Supplement: Supplementary file 1 [file ijms-21-00767-s001.pdf]
